# Supplementary material for: Next-generation diagnostics: virus capture facilitates a sensitive viral diagnosis for epizootic and zoonotic pathogens including SARS-CoV-2
Source: Microbiome. 2021 Feb 20;9:51. doi: 10.1186/s40168-020-00973-z (PMC7896545; doi:10.1186/s40168-020-00973-z)

**Next-generation diagnostics: virus capture facilitates a sensitive viral diagnosis for epizootic and zoonotic pathogens including SARS-CoV-2**

by Wylezich, Calvelage, Schlottau, Ziegler, Pohlmann, Höper & Beer

**Additional file 3: Figure S1**

Coverage of virus genomes generated without (generic HTS) or with enrichment (VirBaits) after reference mapping. Sample and virus names are given as well as references used for mapping analyses with the Genome Sequencer software suite (v. 2.6, default settings; Roche) and displayed using Geneious Prime (2019.2.3).

L03007, Rabies lyssavirus (reference KT336433.1)

**generic HTS** (without enrichment; 0 – 1,630 reads)


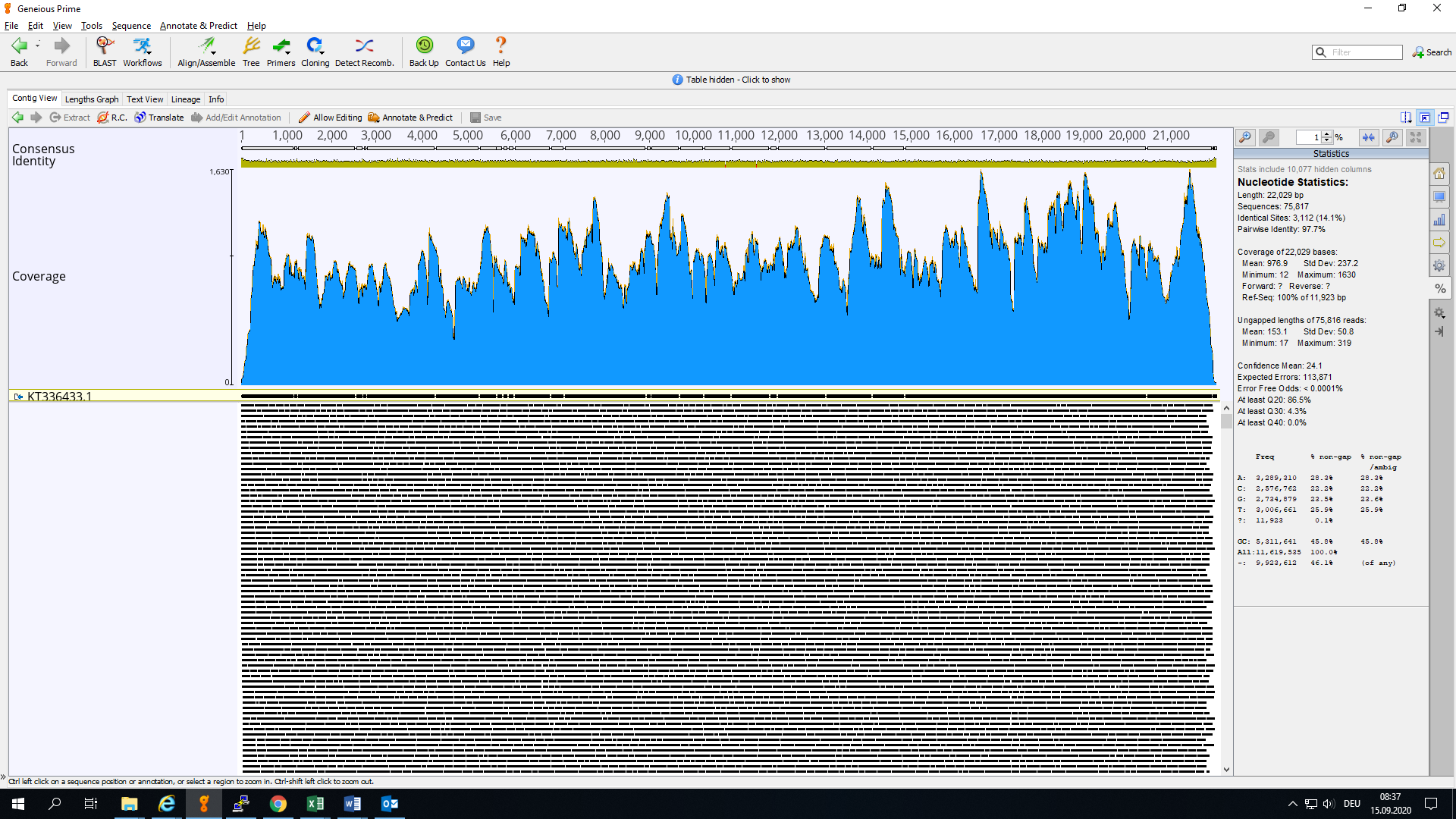


**VirBaits** (with enrichment; 0 – 33,431 reads)


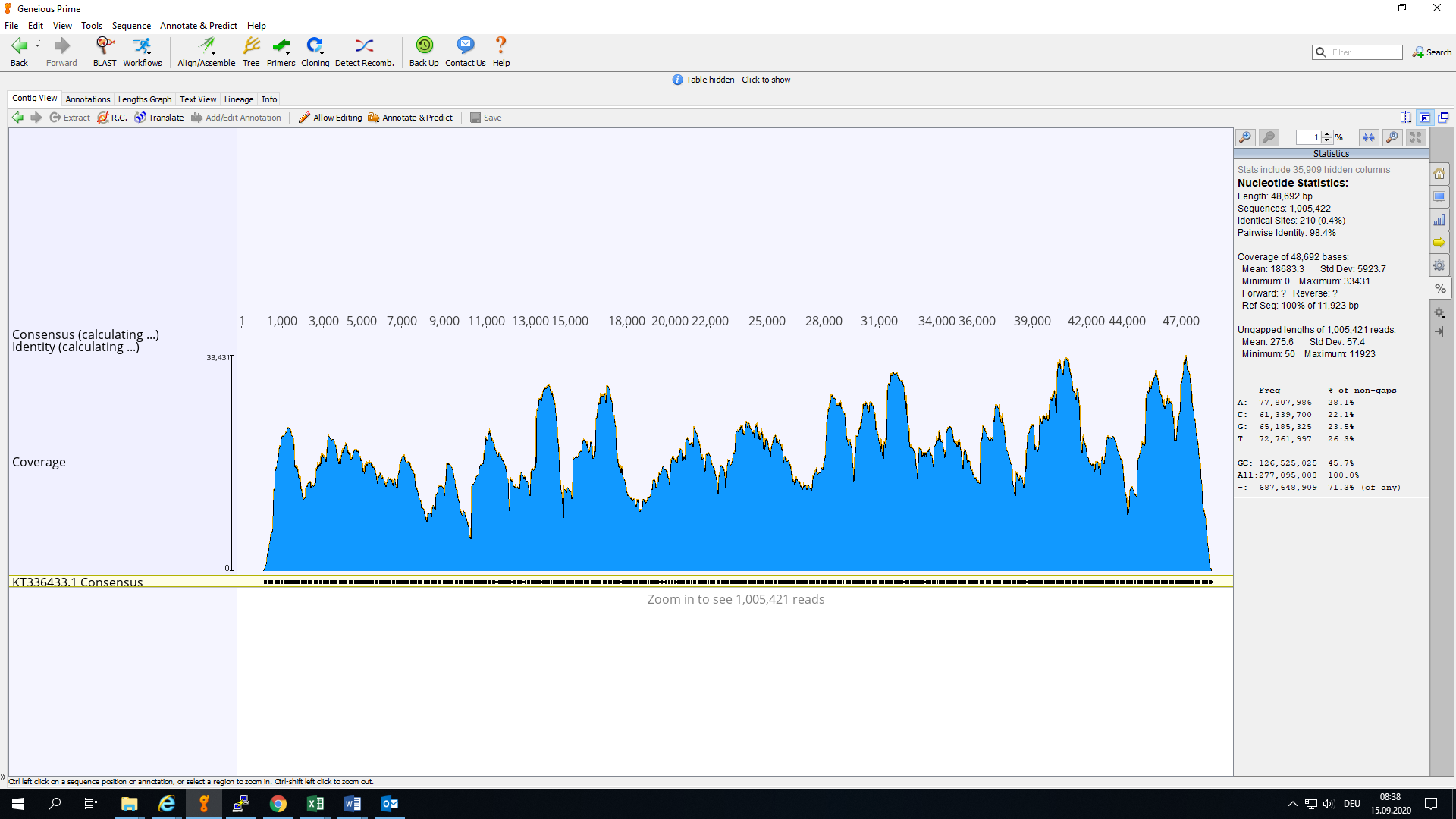


L02762, African swine fever virus (reference LR536725.1)

**generic HTS** (without enrichment; 0 – 110 reads)


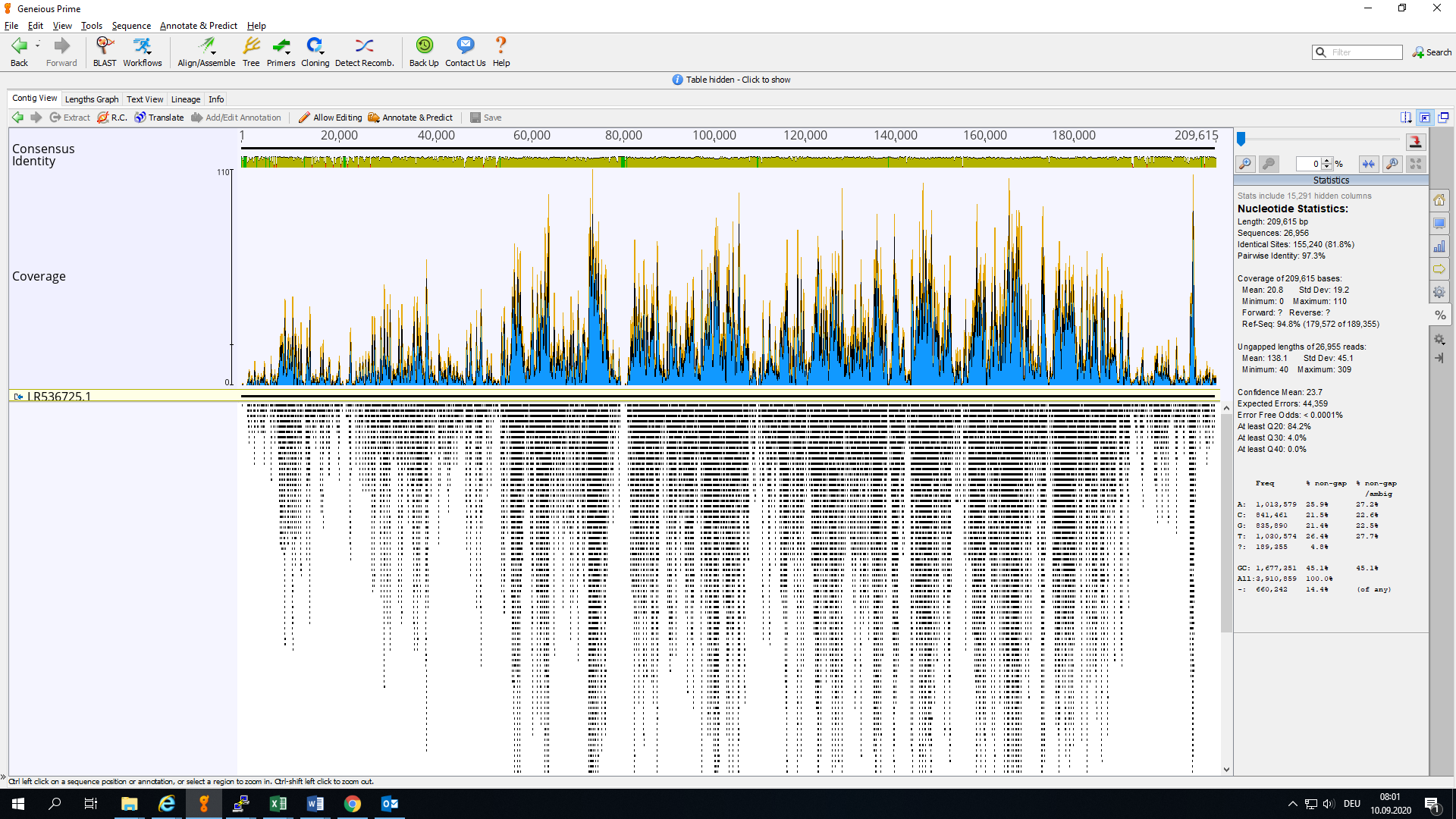


**VirBaits** (with enrichment; 0 – 1,575 reads)


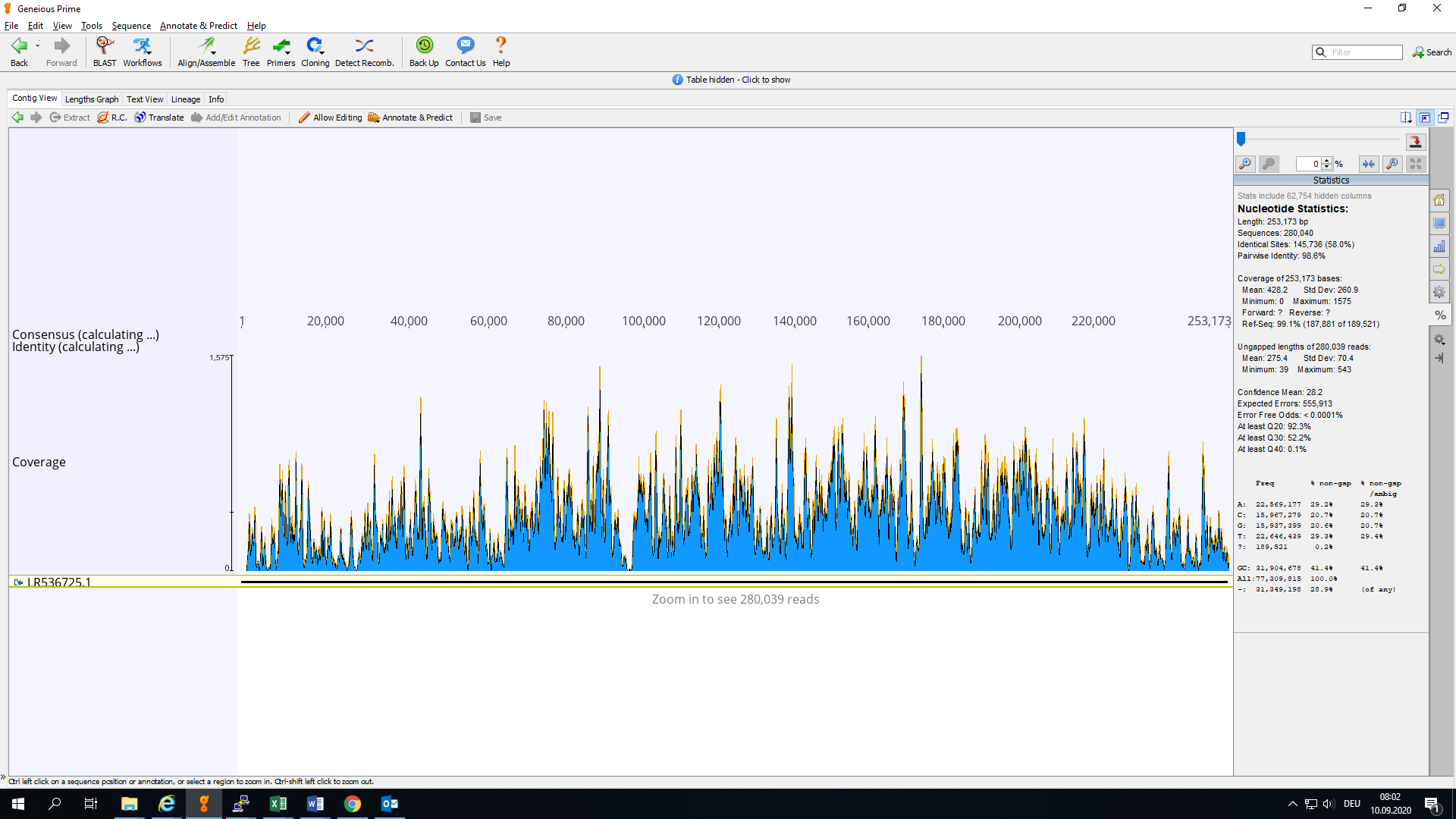


L03451, West Nile virus (reference MH986056.1)

**generic HTS** (without enrichment; 0 – 6 reads)


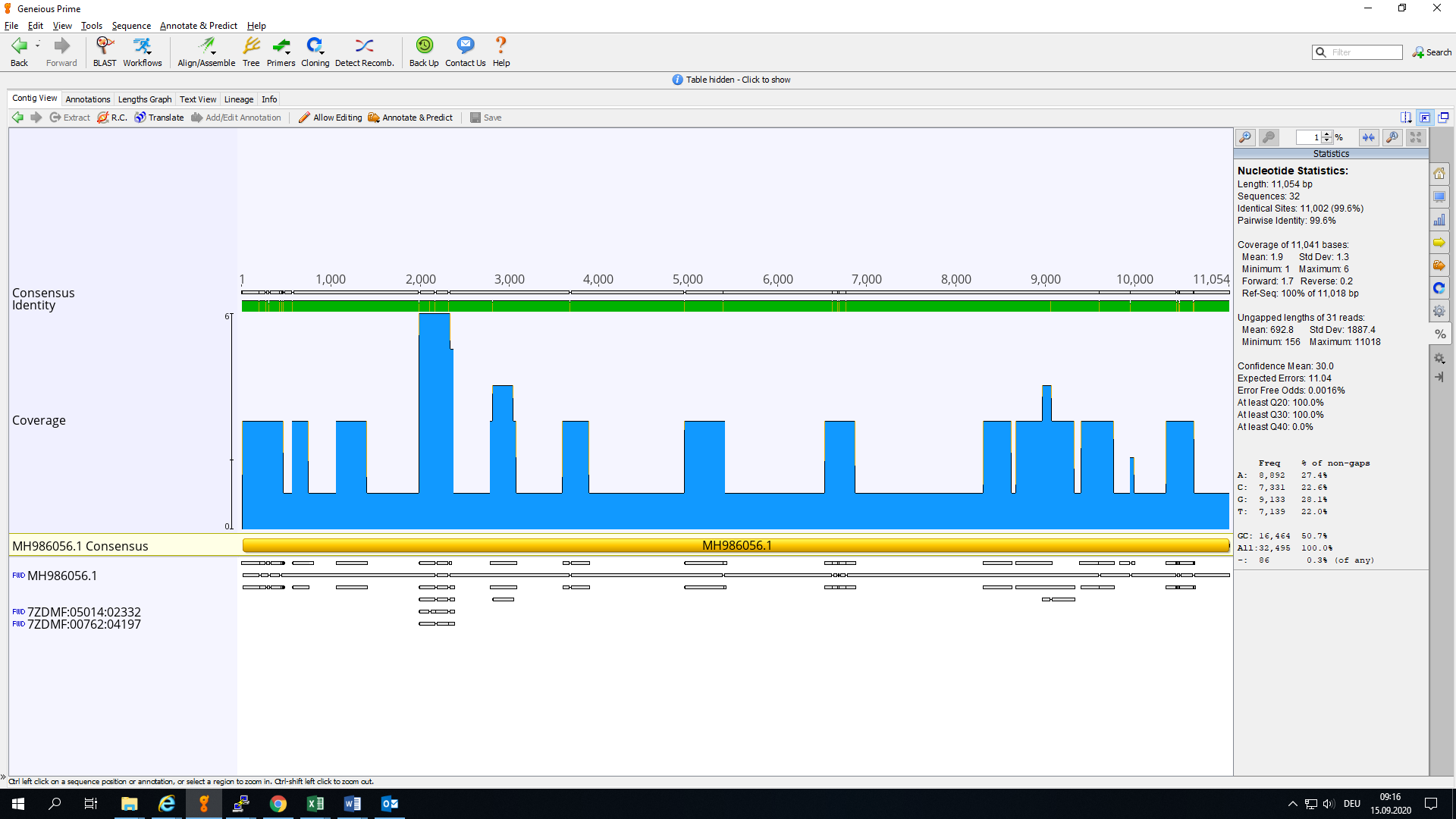


**VirBaits** (with enrichment; 0 – 185 reads)


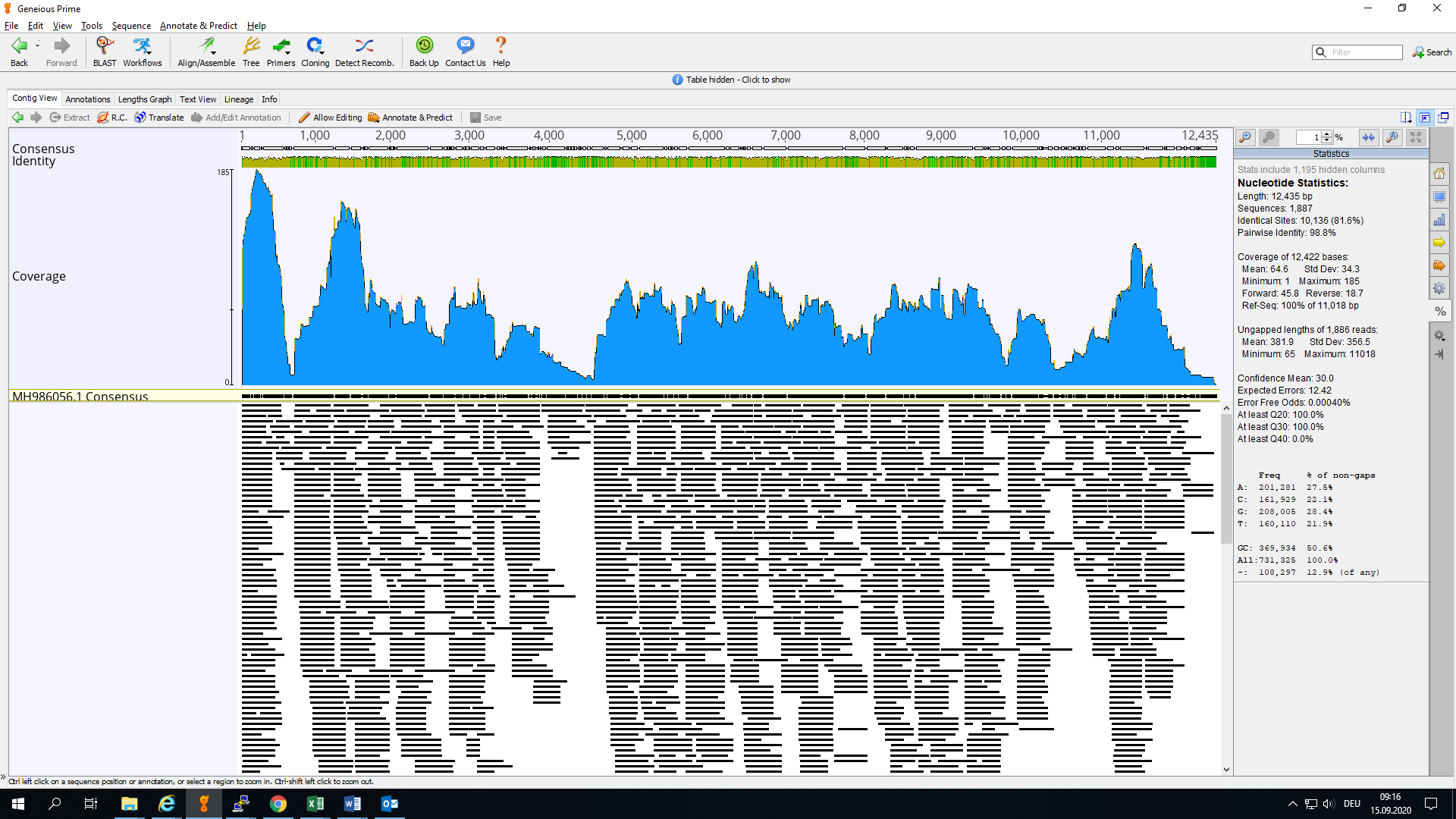


L02838, Rabies lyssavirus (reference KT336433.1)

**generic HTS** (without enrichment; 0 – 3 reads)


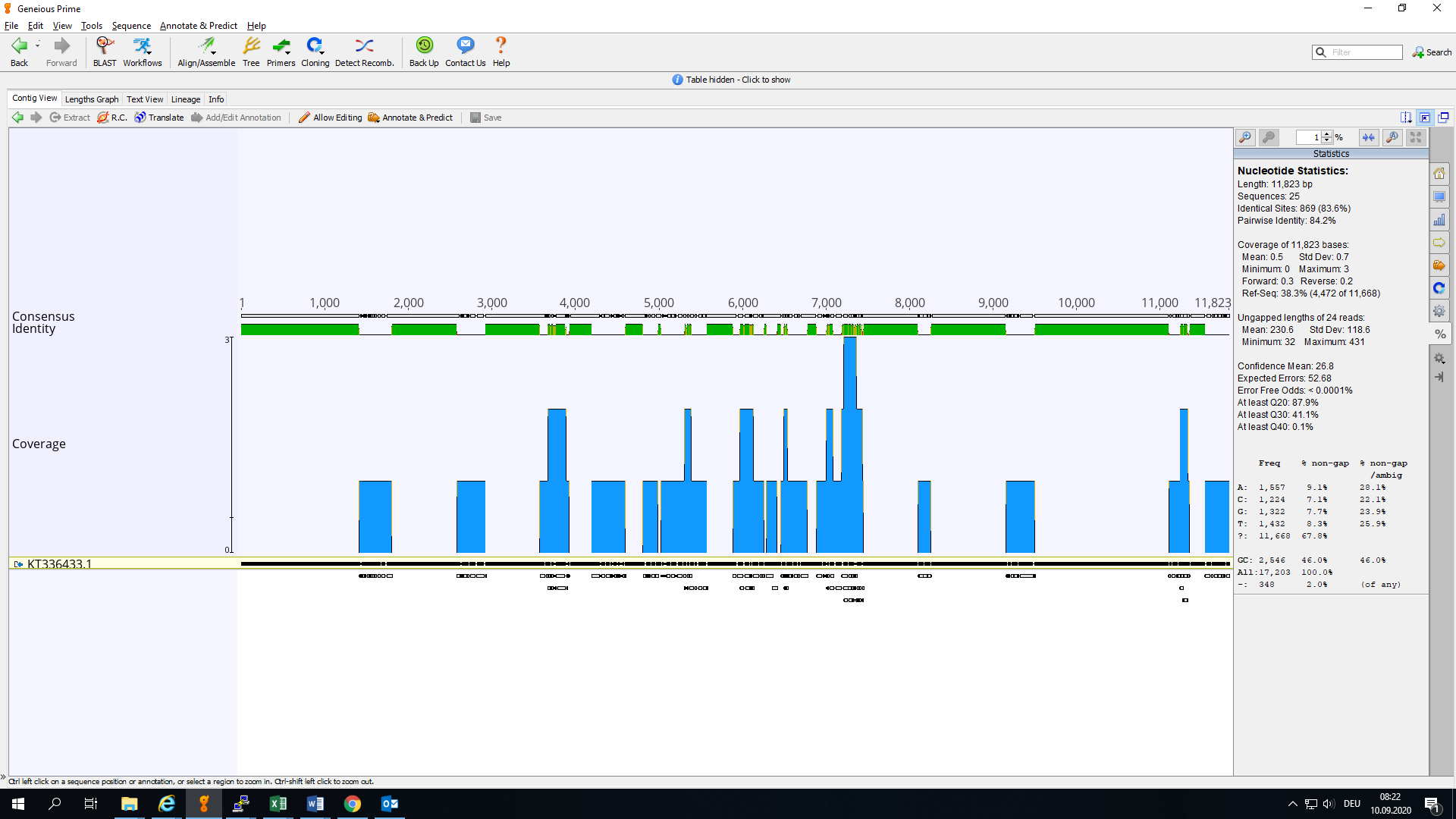


**VirBaits** (with enrichment; 0 – 885 reads)


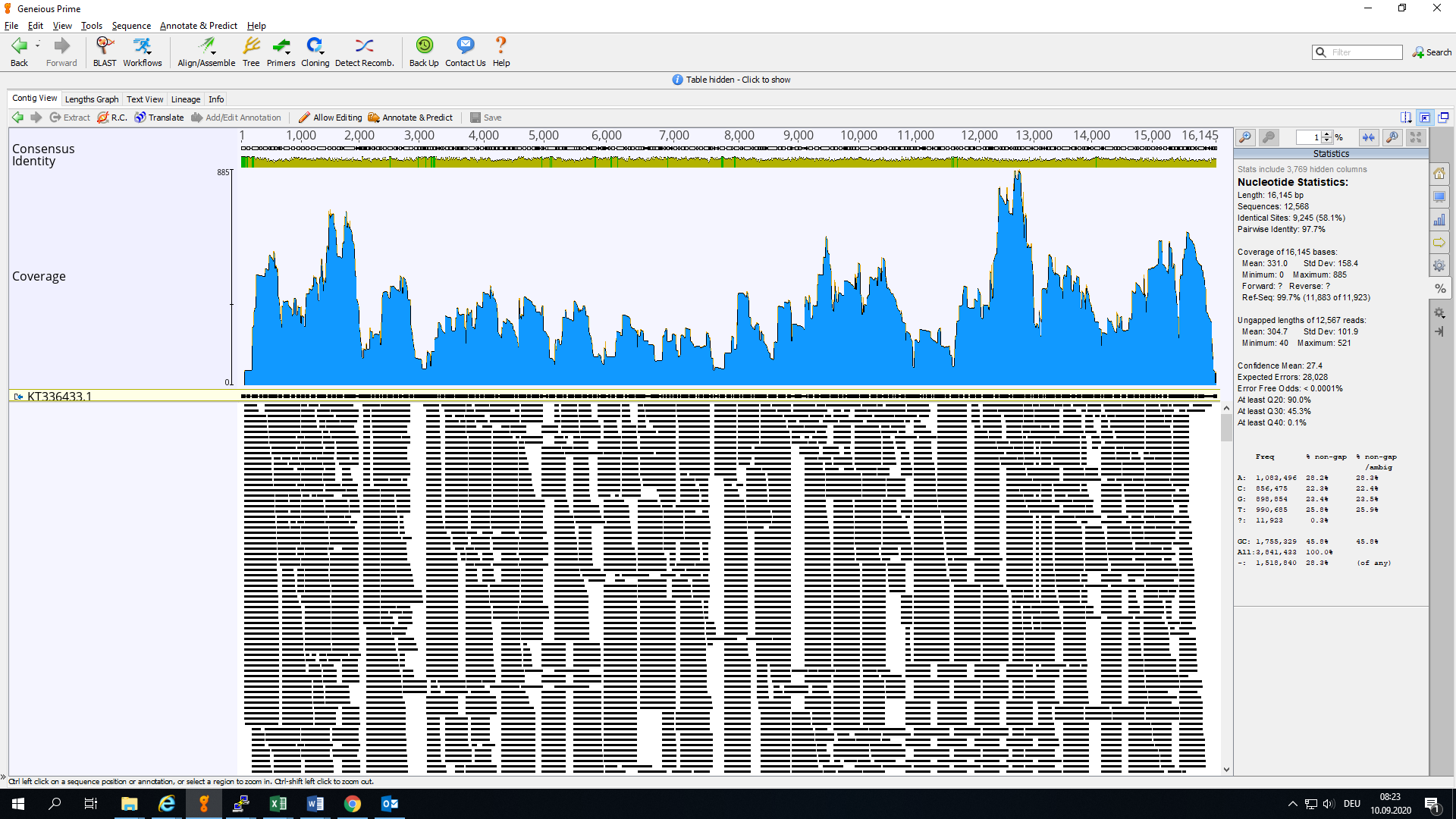


lib02262, ovine picornavirus (reference LR216008.1)

**generic HTS** (without enrichment; 0 – 87 reads)


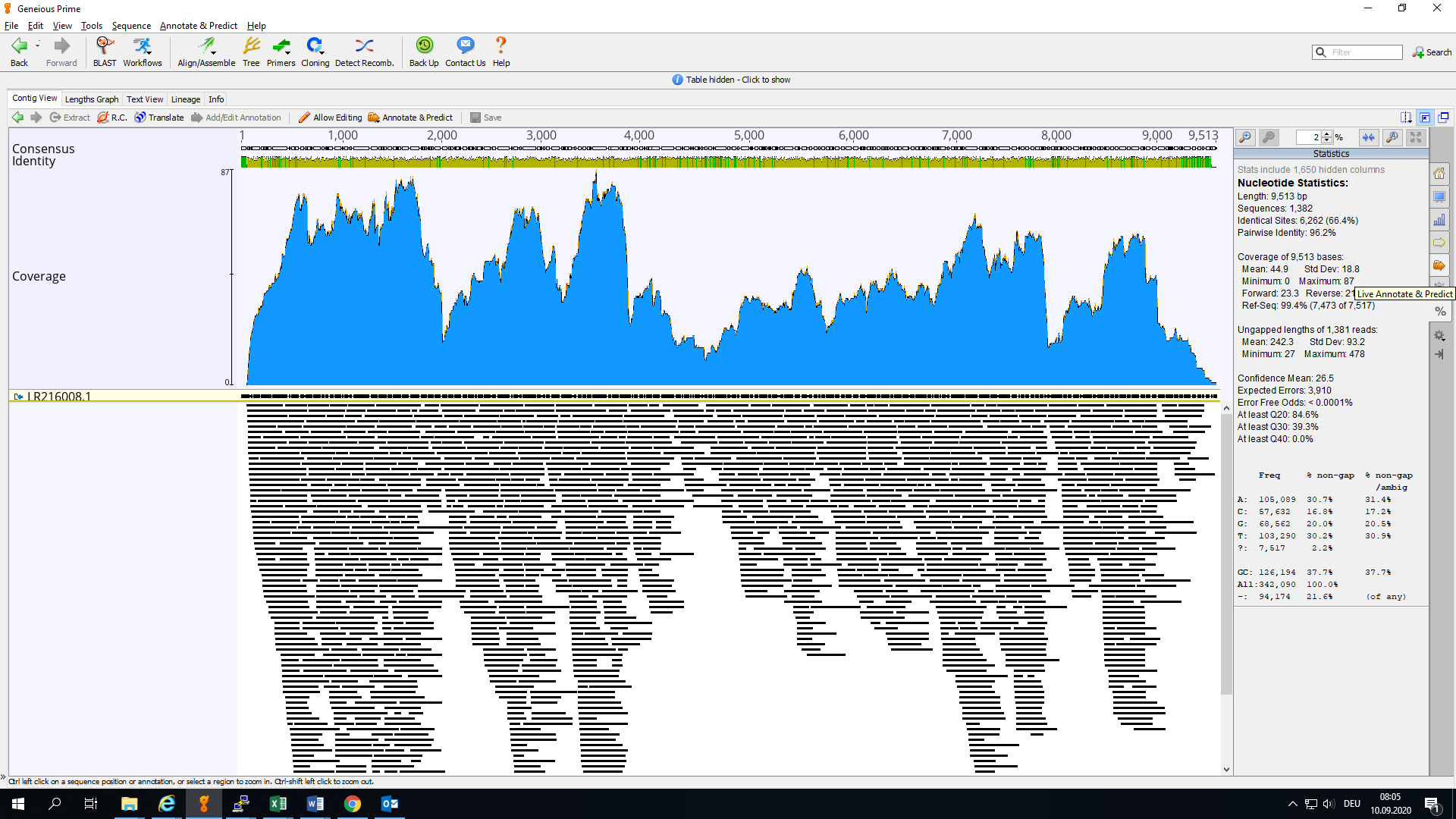


**VirBaits** (with enrichment; 0 – 36 reads)


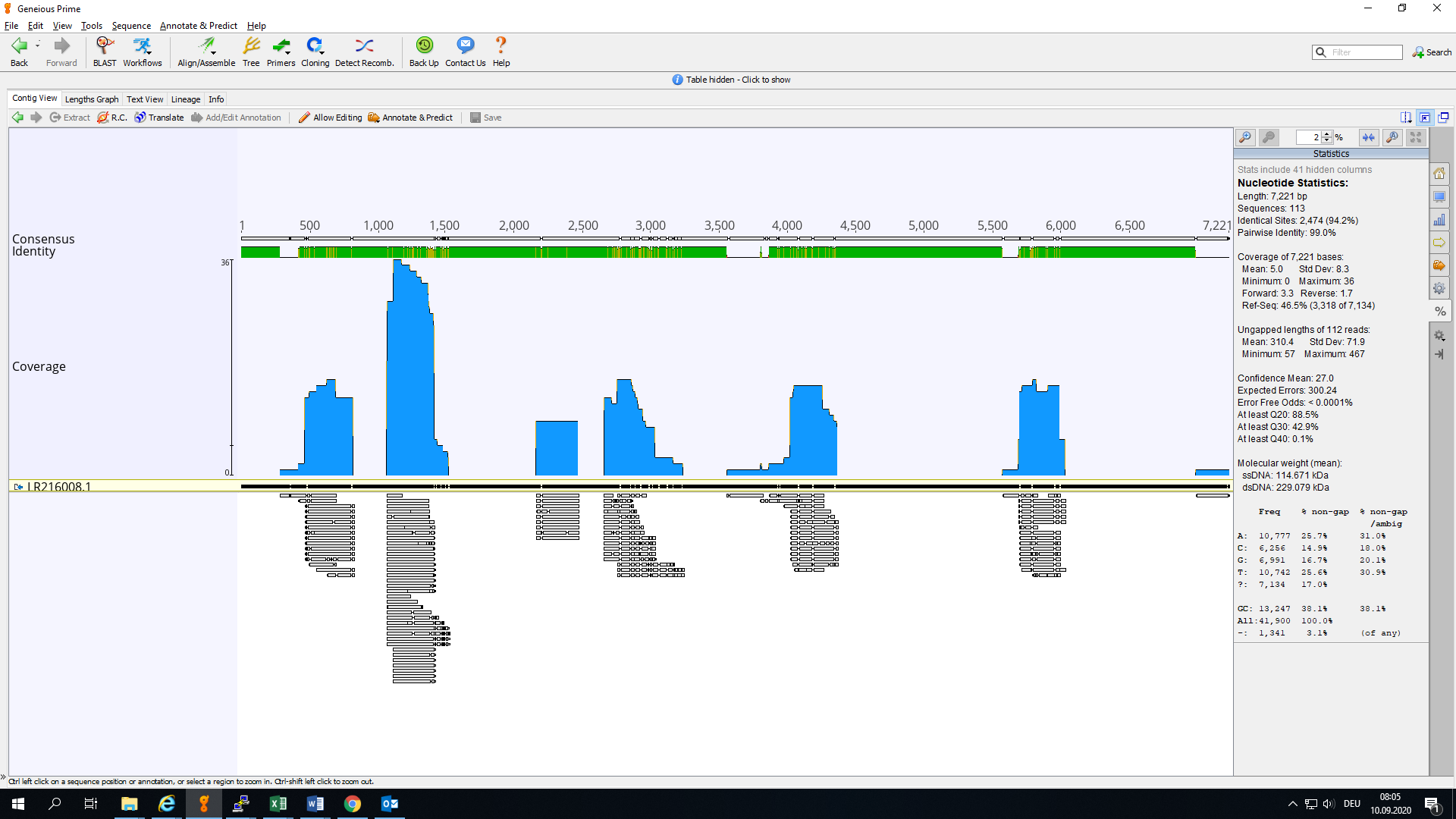

Supplement: Supplementary file 4 — Additional file 3: Figure S1. Coverage of genomes of selected viruses generated without (generic HTS) or with enrichment (VirBaits) after reference mapping with the Genome Sequencer software suite; displayed using Geneious Prime (2019.2.3). [file 40168_2020_973_MOESM4_ESM.docx]
